# Supplementary material for: Locomotor deficits in a mouse model of ALS are paralleled by loss of V1-interneuron connections onto fast motor neurons
Source: Nat Commun. 2021 May 31;12:3251. doi: 10.1038/s41467-021-23224-7 (PMC8166981; doi:10.1038/s41467-021-23224-7)
Supplement: Supplementary file 1 — Supplementary Information [file 41467_2021_23224_MOESM1_ESM.pdf]

Supplementary Information

Locomotor deficits in a mouse model of ALS are paralleled by loss of V1-interneuron connections onto fast motor neurons

Ilary Allodi, Roser Montañana-Rosell, Raghavendra Selvan, Peter Löw, and Ole Kiehn

Contents:

- Supplementary Figures 1-6
- Supplementary Tables 1-2
- Supplementary References

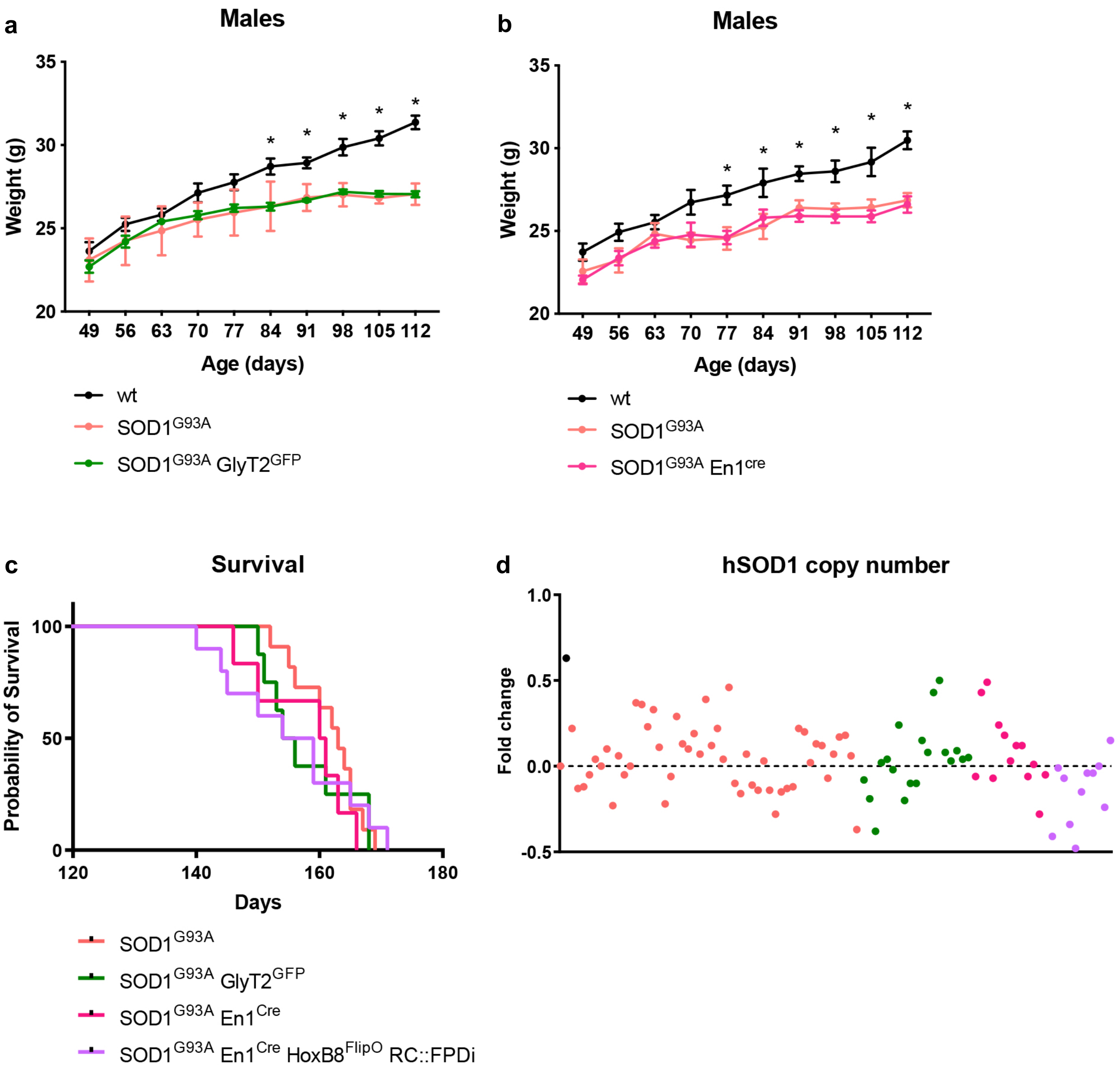

Supplementary figure 1. (a) Weights of SOD1^{G93A} and SOD1^{G93A};GlyT2^{GFP} males compared to wild-type mice. After crossing, SOD1^{G93A};GlyT2^{GFP} mice do not differ from the SOD1^{G93A} mice but both strains differ from wild-type (wt) littermates (two-way ANOVA and Dunnett's post hoc, F(18, 168)=1.733, P84 SOD1^{G93A} P=0.0244, P84 SOD1^{G93A};GlyT2^{GFP} P=0.0058; wt N=11 mice, SOD1^{G93A} N=6 mice, SOD1^{G93A};GlyT2^{GFP} N=8 mice). (b) Weights of SOD1^{G93A} and SOD1^{G93A};En1^{cre} males compared to wild-type mice. SOD1^{G93A} and SOD1^{G93A};En1^{cre} weights do not differ between each other, but they differ from wild-type littermates (two-way ANOVA and Dunnett's post hoc, F(18, 260)=0.9744, P77 SOD1^{G93A} P=0.0213, P77 SOD1^{G93A};En1^{cre} P=0.0127; wt N=18 mice, SOD1^{G93A} N=12 mice, SOD1^{G93A};En1^{cre} N=19 mice). (c) Kaplan Meyer survival curve comparing all the SOD1^{G93A} crossings utilized in the study to the congenic SOD1^{G93A} strain. No significant differences were observed among the conditions (two-tailed Log-rank (Mantel-Cox) test, P=0.8586, df=3, Chi square=0.7616; SOD1^{G93A} N= 11 mice, SOD1^{G93A};GlyT2^{GFP} N= 8 mice, SOD1^{G93A};En1^{cre} N=6 mice, SOD1^{G93A}; En1^{cre};HoxB8^{FlipO};RC::Di N= 10 mice). (d) Copy number of human SOD1 mutations carried by all the animals included in the study. Orange dots depict fold change for SOD1^{G93A} mice, green dots SOD1^{G93A};GlyT2^{GFP}, magenta dots SOD1^{G93A};En1^{cre} and lilac dots SOD1^{G93A};En1^{cre};HoxB8^{FlipO};RC::Di mice. First orange dot is the positive control - SOD1^{G93A} founder carrying 25 copies of the mutated gene, second grey dot the negative control - SOD1^{127X} carrying 19 copies of the mutated gene. In all graphs, data are presented as mean values ± SEM. Source data are provided as a Source Data file.

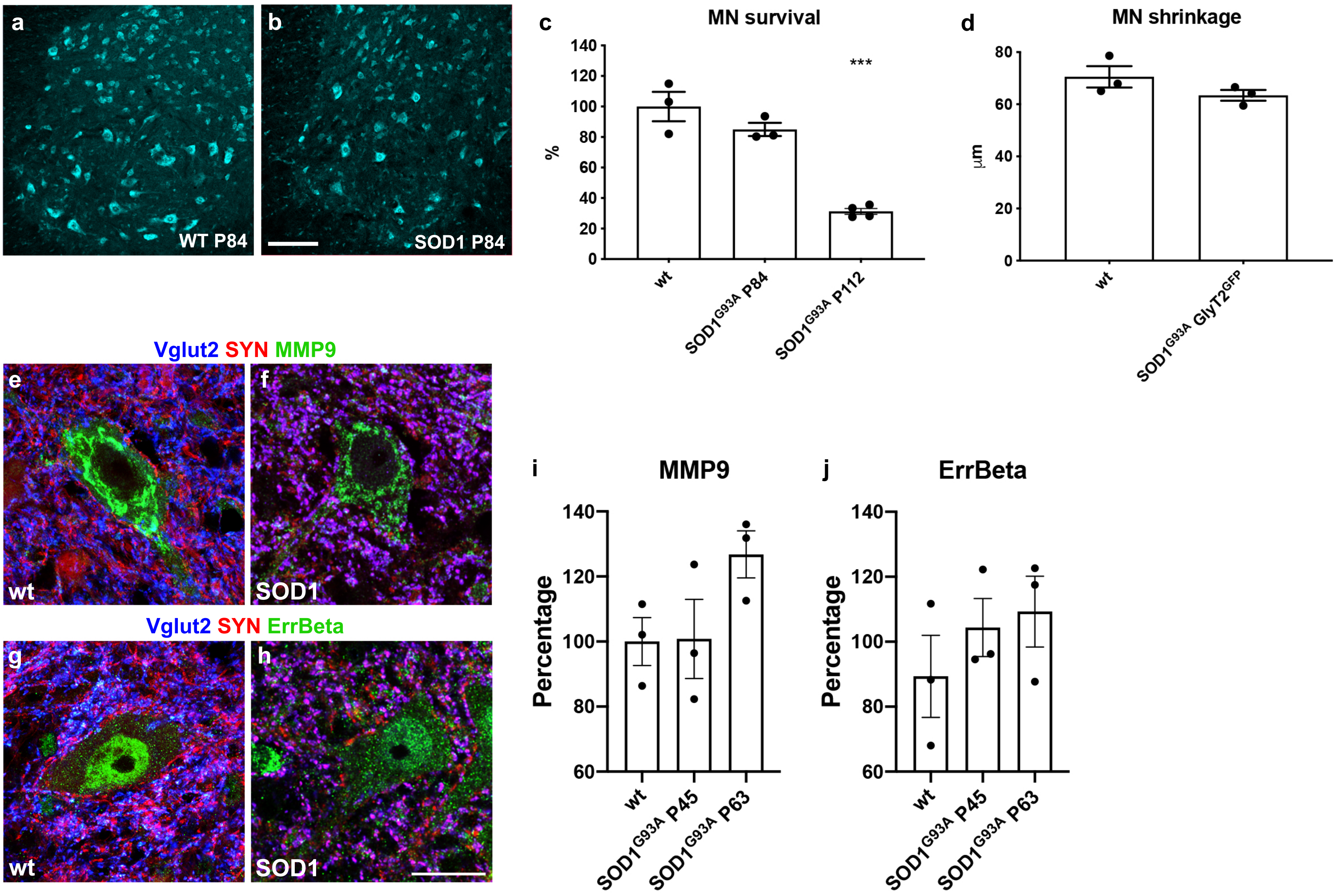

Supplementary figure 2. (a-b) Motor neuron quantification is performed utilizing Fluor Nissl staining at P84 and P112 timepoints and compared to wild-type (wt) littermates in SOD1^{G93A};GlyT2^{GFP} mice. (c) The number of motor neurons detected per hemicord in control mice was 15.4±3.4 (N=3 mice) while 12.±10.7 (N=3 mice) at P84 and 5.4±0.8 (N=4 mice) at P112. Between 10 and 12 hemicords were quantified per mouse per condition. Differences in motor neuron survival were statistically significant at P112 timepoint (one-way ANOVA and Dunnett's post hoc, F(2, 7)=45.95, P=0.000094). (d) Differences in synaptic densities was not due to changes in soma area until P84, since motor neuron shrinkage was not detected at this timepoint (two-tailed t test, P=0.1980, t=1.542, df=4; N=3 mice). Fluor Nissl in cyan, scale bar in (b) = 100 µm also representative of (a). (e-j) Glutamatergic buttons present on fast and slow motor neuron somata at P45 and P63. (e-f) Microphotographs show Vglut2 positive buttons onto MMP9^{+} neurons in wild-type and P63 SOD1^{G93A} mice. MMP9 in green, synaptophysin (SYN) in red, Vglut2 in blue. (i) Vglut2 button density does not differ among conditions at these timepoints (one-way ANOVA, F(2, 6)=2.748, P=0.1422; N=3 mice per condition). (g-h) ErrBeta^{+} motor neurons in control and P63 SOD1^{G93A} mice, respectively. ErrBeta in green, SYN in red, Vglut2 in blue. (j) Quantifications of Vglut2^{+} buttons in control, P45 and P63 mice show no changes in density (one-way ANOVA, F(2, 6)=0.9038, P=0.4538; N=3 mice per condition). Scale bar in (h) = 50 µm representative of images in (e-h). In all graphs, data are presented as mean values ± SEM. Source data are provided as a Source Data file.

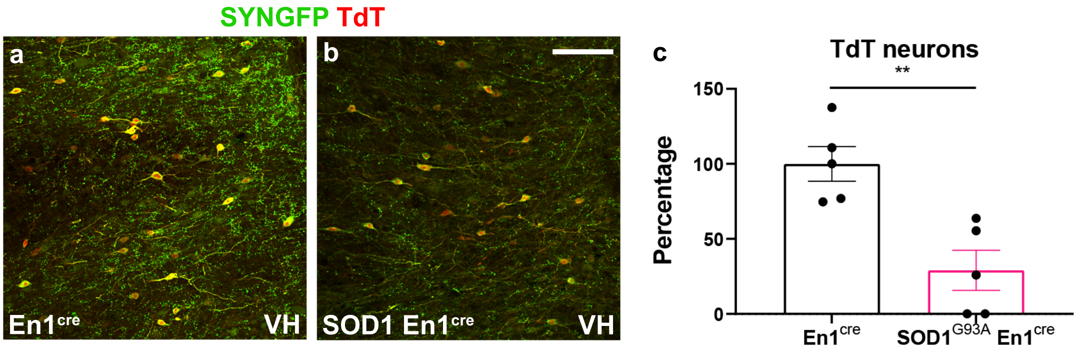

Supplementary figure 3. Characterization of $SOD1^{G93A}$;$En1^{cre}$ animals. Microphotographs depicting spinal cords after injection in $En1^{cre}$ mice (a) and in $SOD1^{G93A}$;$En1^{cre}$ animals (b). (c) Percentage of TdT positive interneurons three weeks after viral delivery, number of positive neurons is significantly lower in $SOD1^{G93A}$;$En1^{cre}$ than in $En1^{cre}$ mice ($En1^{cre}$ = 99±11.62%; $En1^{cre}$;$SOD1^{G93A}$ = 29.03±13.41%; two-tailed t test P=0.0040, t=3.999, df=8; N=5 independent mice per condition). Scale bar in (b) = 100 µm also representative of (a). Data are presented as mean values ± SEM in (c). Source data are provided as a Source Data file.

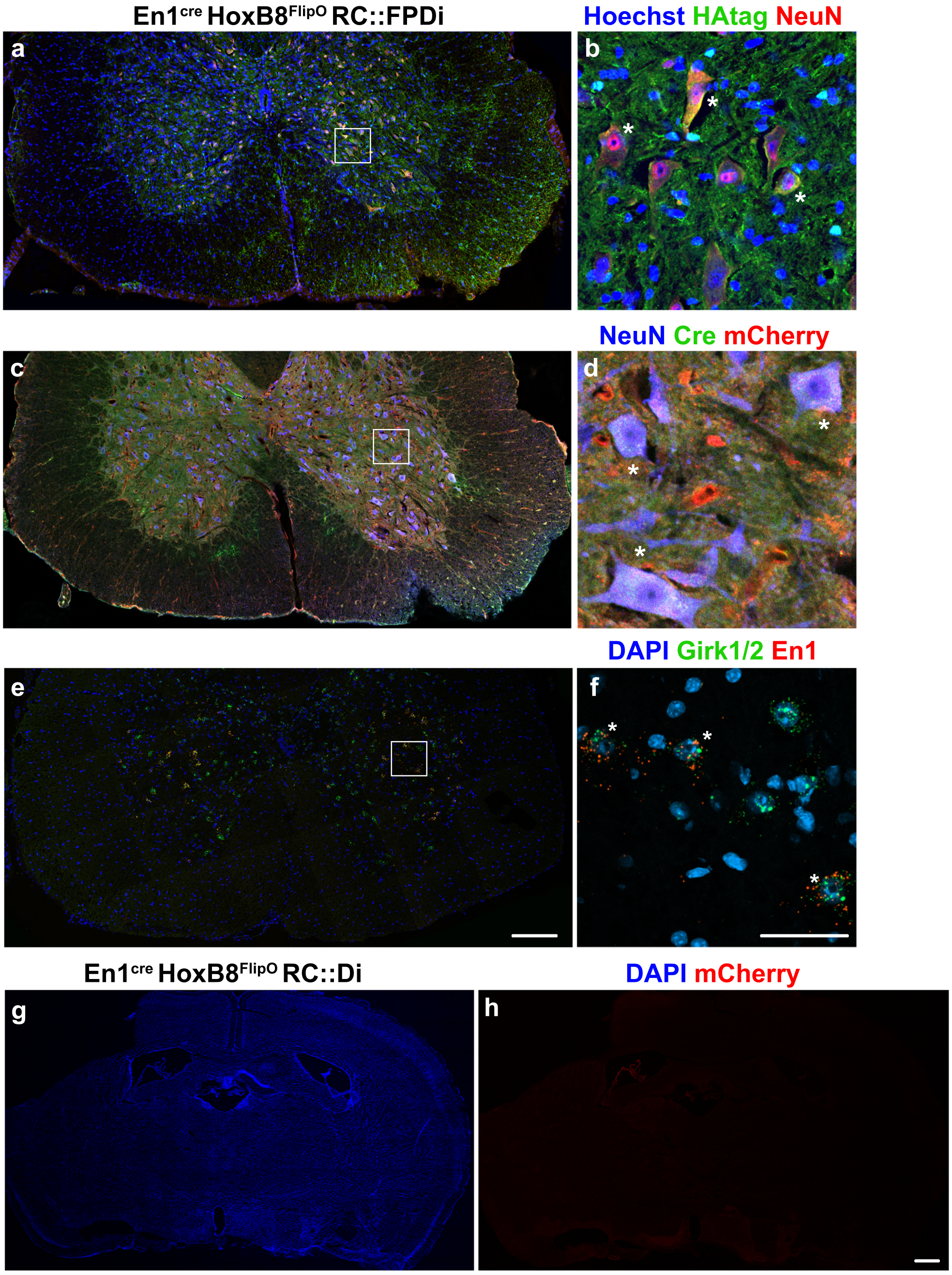

Supplementary figure 4. Characterization of En1[cre];HoxB8[FlipO];RC::Di mice. (a) Dual recombinase of (F; Flt) (P; LoxP) results in HA-tag expression in En1[cre];HoxB8[FlipO];RC::Di positive neurons, higher magnification in (b). HA-tag in green, NeuN in red and Hoechst in blue. (c) Ventral horn of the spinal cord of an En1[cre];HoxB8[FlipO];RC::Di mouse showing mCherry expression (red) after dual recombinase. Cre+ (green) and NeuN (blue) positive neurons co-label with mCherry and are marked with *. Experiments in (a-d) were repeated in 3 independent mice. (e) Girk1 and Girk2 expression (green) analyzed by RNAscope in situ hybridization in En1 positive neurons (orange) confirms the presence of the necessary channels for DREADD inhibition in the neuronal population of interest. DAPI in blue, magnification is shown in (f). Girk expression was analyzed in 7 independent mice upon CNO administration. Scale bar in (e) = 200 μm representative also of (a) and (c). Scale bar in (f) = 50 μm representative also of (b) and (d). (g-h) En1[cre];HoxB8[FlipO];RC::Di mice do not express mCherry in the brain, corroborating the spinal restricted silencing of En1 neurons. DAPI in blue (g) and mCherry in red (h). mCherry expression in the brain was assessed in 2 independent mice. Scale bar in (h) = 500 μm representative also of (g).

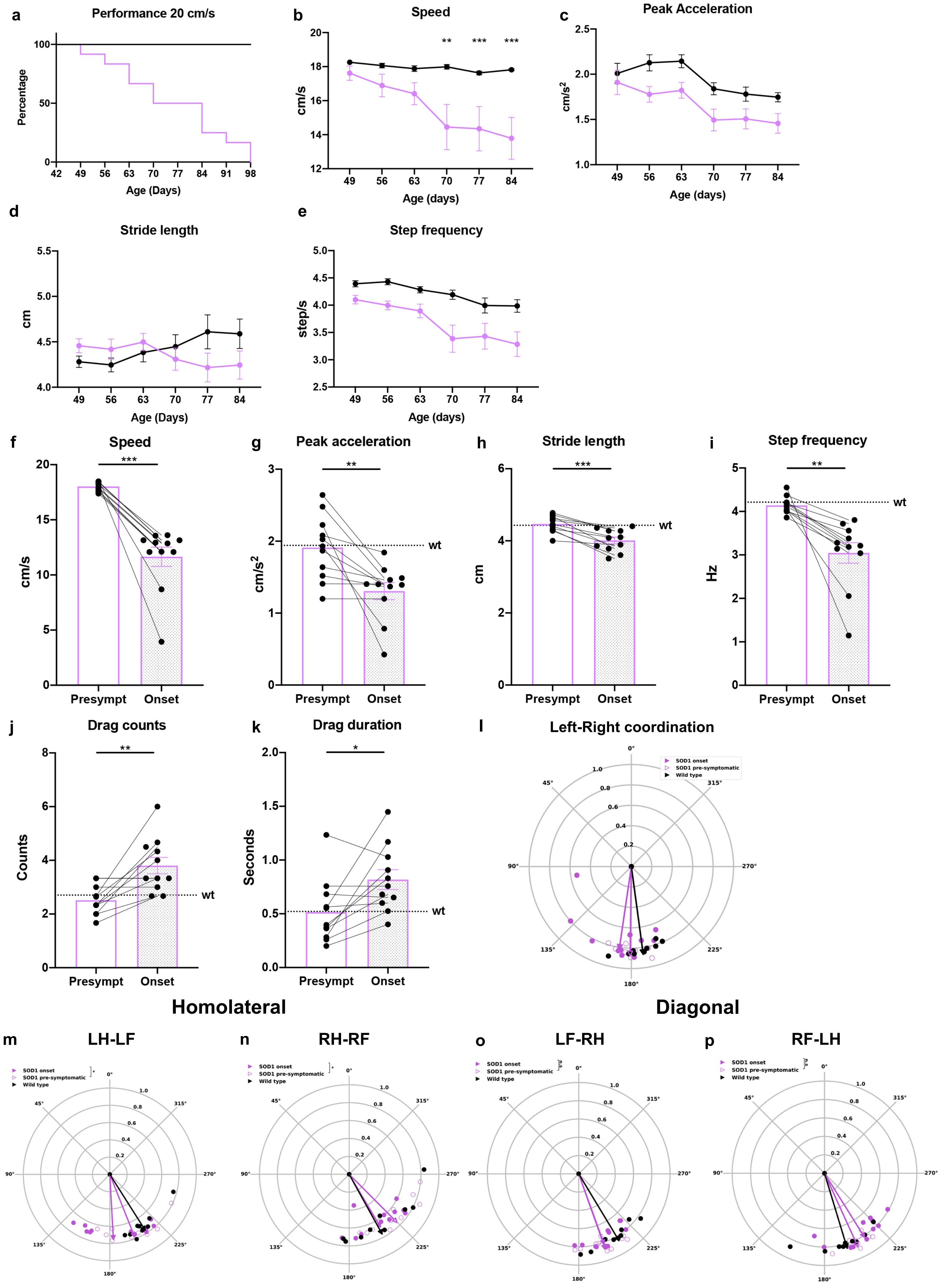

Supplementary figure 5

Supplementary figure 5. Characterization of Onset of locomotor phenotype in $SOD1^{G93A}$;$En1^{cre}$;$HoxB8^{FlipO}$;RC::Di mice. (a) Phenotype validation of the $SOD1^{G93A}$;$En1^{cre}$;$HoxB8^{FlipO}$;RC::Di showed similar phenotype as $SOD1^{G93A}$ mice on the treadmill (median=77; two-tailed Gehan-Breslow-Wilcoxon test, P=0.00002, df=1, Chi square=18.17; N=11 mice). The locomotor phenotype included progressive loss of speed (b) (two-way ANOVA and Dunnett's post hoc, F(5, 90)=2.626, P70 P=0.0013, P77 P=0.0009, P84 P=0.00009; wild-type (wt) N=9 mice, $SOD1^{G93A}$;$En1^{cre}$;$HoxB8^{FlipO}$;RC::Di N=11 mice), reduced peak acceleration (c) (two-way ANOVA, F(5, 90)=0.5017, P=0.0013; wt N=9 mice, $SOD1^{G93A}$;$En1^{cre}$;$HoxB8^{FlipO}$;RC::Di N=11 mice), decrease in stride length (d) (two-way ANOVA, F(5, 90)=3.261, P=0.0094; wt N=9 mice, $SOD1^{G93A}$;$En1^{cre}$;$HoxB8^{FlipO}$;RC::Di N=11 mice) and in step frequency (e) (two-way ANOVA, F(5, 90)=1.080, P=0.0008; wt N=9 mice, $SOD1^{G93A}$;$En1^{cre}$;$HoxB8^{FlipO}$;RC::Di N=11 mice). As for the $SOD1^{G93A}$ mice, the Onset of locomotor phenotype in $SOD1^{G93A}$;$En1^{cre}$;$HoxB8^{FlipO}$;RC::Di mice was characterized by decrease in speed of locomotion (f) (two-tailed t test P=0.00009, t=6.665, df=9; N=11 mice before and after onset), reduced peak acceleration (g) (two-tailed t test P=0.0060, t=3.471, df=10; N=11 mice), decreased stride length (h) (two-tailed t test P=0.0002, t=5.860, df=10; N=11 mice) and decreased step frequency (i) (two-tailed t test P=0.0020, t=4.158. df=10; N=11 mice). Dragging events were increased both in number (j) (two-tailed t test, P= 0.0034, t=3.815, df=10; N=11 mice) and duration (k) (two-tailed t test, P= 0.0195, t=2.778, df=10; N=11 mice). (l) Left-right coordination remained unchanged (two-tailed Watson-Williams test, pre-symptomatic vs onset P=0.2792; N=11 mice, n=15 steps per mouse). Homolateral coordination shown in (m) and (n) shifted slightly but significantly after Onset of locomotor phenotype as observed in the $SOD1^{G93A}$ mice (two-tailed Watson-Williams test, homolateral LH-LF P=0.0097, RH-RF P=0.0216; N=11 mice, n=15 steps per mouse) while the diagonal coordination (o-p) does not differ from the pre-symptomatic stages (RF-LH P=0.2389, LF-RH P=0.2376; wt N=9 mice, $SOD1^{G93A}$;$En1^{cre}$;$HoxB8^{FlipO}$;RC::Di N=11 mice before and after onset, n=15 steps per mouse). Magenta-full = onset; magenta empty = pre-symptomatic; black dots = wild-type. Dotted lines show averages for wild-type (wt) animals in all parameters included in the analysis. In all graphs, data are presented as mean values ± SEM. Source data are provided as a Source Data file.

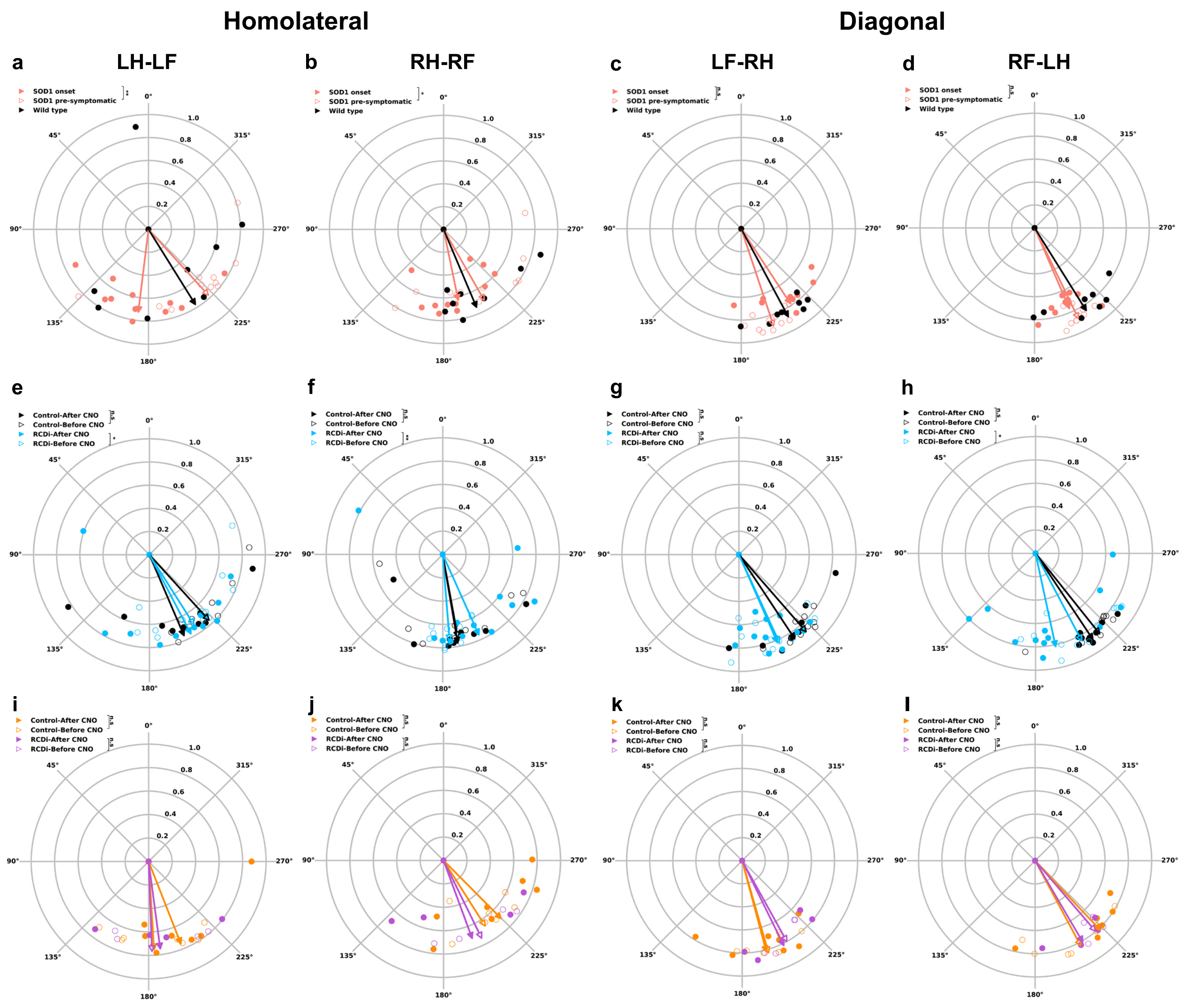

Supplementary figure 6. Kinematic analysis showing homolateral and diagonal limb coordination in SOD1^{G93A} compared to En1^{cre};HoxB8^{FlipO};RC::Di and SOD1^{G93A};En1^{cre};HoxB8^{FlipO};RC::Di mice. (a-b) Homolateral coordination of the limbs after Onset of locomotor phenotype differs from pre-symptomatic stages (two-tailed Watson-Williams test, LH-LF P=0.00009, RH-RF P=0.0462; wt N=8 mice, SOD1^{G93A} N=11 mice before and after onset, n=15 steps per mouse). No changes are observed in the diagonal coordination (two-tailed Watson-Williams test, LF-RH P=0.0880, RF-LH P=0.8006; wt N=8 mice, SOD1^{G93A} N=11 mice, n=15 steps per mouse) (c-d). Similar changes are observed in En1^{cre};HoxB8^{FlipO};RC::Di after CNO administration. Shift in the homolateral coordination in (e-f) (two-tailed Watson-Williams test, LH-LF P=0.0481, RH-RF P=0.00009; control N=9 mice, En1^{cre};HoxB8^{FlipO};RC::Di N=12 mice before and after CNO administration, n=15 steps per mouse), while the diagonal coordination (g-h) is less affected by the En1 silencing (two-tailed Watson-Williams test, LF-RH P=0.8453, RF-LH P=0.0059; control N=12 mice, En1^{cre};HoxB8^{FlipO};RC::Di N=12 mice, n=15 steps per mouse). No changes could be observed after CNO administration in the SOD1^{G93A};En1^{cre};HoxB8^{FlipO};RC::Di mice once they reached the Onset of locomotor phenotype. Homolateral coordination in (i-j) (two-tailed Watson-Williams test, LH-LF P=0.5051, RH-RF P=0.1575; control N=7 mice, quadruple transgenics N=4 mice before and after CNO administration, n=15 steps per mouse) and diagonal coordination in (k-l) (two-tailed Watson-Williams test, RF-LH P=0.1608, LF-RH P=0.2757; control N=7 and quadruple transgenics N=4 mice before and after CNO administration, n=15 steps per mouse). Source data are provided as a Source Data file.

Supplementary figure 6

Supplementary table 1. Primary antibody list.

Application	Target	Source & catalogue nr.	Host species	Concentration	Reference
NMJ quantification	α-Bungarotoxin Alexa Fluor 488	Invitrogen B13422		1:500	[1, 2]
NMJ quantification	Neurofilament (165 kDa)	DSHB (2H3)	Mouse	1:50	[1, 2]
NMJ quantification	SV2A	DSHB (SV2)	Mouse	1:50	[1, 2]
Synaptic density	GFP	Abcam Ab-13970	Chicken	1:1000	[3]
FF MN Identification	MMP-9	Sigma Aldrich M9570-100UG	Goat	1:1000	[4]
S MN Identification	ErrBeta	R&D Systems PP-H6705-00	Mouse	1:500	[5]
Synaptic density	Synaptophysin Alexa Fluor 594	Santa Cruz Sc-17750	Mouse	1:100	[6]
Synaptic density	DsRed	Clontech 632496	Rabbit	1:1000	[7]
Synaptic density	VGLUT2	Synaptic Systems #135404	Guinea-pig	1:1000	[8]
Intersectional expression	HA-tag	Sigma Aldrich H6908	Rabbit	1:100	[9]
Intersectional expression	NeuN	Millipore ABN91	Chicken	1:1000	[10]

All primary antibodies have been extensively used and evaluated for specificity in many previous publications as indicated in the Antibody Registry - https://antibodyregistry.org and from the manufacturers' homepages (Invitrogen, DSHB, Abcam, Sigma Aldrich, R&D Systems, Santa Cruz, Clontech, Synaptic Systems, Millipore). References are given to a few studies.

Supplementary table 2. RNAscope *in situ* hybridization probes.

**Application**	**Target**	**Channel**	**Source & catalogue nr.**	**Fluorophore combination**
En1 expression & GIRK control	En1	C1	ACD Bio-techne 442651	Opal^{TM} 570
ChAT expression	ChAT	C2	ACD Bio-techne 408731-C2	Opal^{TM} 520
GIRK expression	Girk1 (Kcnj3)	C2	ACD Bio-techne 523951-C2	Opal^{TM} 520
GIRK expression	Girk2 (Kcnj6)	C3	ACD Bio-techne 472321-C3	Opal^{TM} 520

All probes have been extensively evaluated for specificity by the manufacturers (Advanced Cell Diagnostics, Bio-techne).

Supplementary References

1. Allodi I*, et al.* Differential neuronal vulnerability identifies IGF-2 as a protective factor in ALS. *Sci Rep* **6**, 25960 (2016).

2. Comley LH, Nijssen J, Frost-Nylen J, Hedlund E. Cross-disease comparison of amyotrophic lateral sclerosis and spinal muscular atrophy reveals conservation of selective vulnerability but differential neuromuscular junction pathology. *J Comp Neurol* **524**, 1424-1442 (2016).

3. Allodi I*, et al.* Modeling Motor Neuron Resilience in ALS Using Stem Cells. *Stem Cell Reports* **12**, 1329-1341 (2019).

4. Kaplan A*, et al.* Neuronal matrix metalloproteinase-9 is a determinant of selective neurodegeneration. *Neuron* **81**, 333-348 (2014).

5. Enjin A*, et al.* Identification of novel spinal cholinergic genetic subtypes disclose Chodl and Pitx2 as markers for fast motor neurons and partition cells. *J Comp Neurol* **518**, 2284-2304 (2010).

6. Luo J*, et al.* LncRNA-p21 alters the antiandrogen enzalutamide-induced prostate cancer neuroendocrine differentiation via modulating the EZH2/STAT3 signaling. *Nat Commun* **10**, 2571 (2019).

7. Caggiano V*, et al.* Midbrain circuits that set locomotor speed and gait selection. *Nature* **553**, 455-460 (2018).

8. Ni Y*, et al.* Characterization of long descending premotor propriospinal neurons in the spinal cord. *J Neurosci* **34**, 9404-9417 (2014).

9. Li L*, et al.* Pathological Alterations of Tau in Alzheimer's Disease and 3xTg-AD Mouse Brains. *Mol Neurobiol* **56**, 6168-6183 (2019).

10. Magno LAV*, et al.* Contribution of neuronal calcium sensor 1 (Ncs-1) to anxiolytic-like and social behavior mediated by valproate and Gsk3 inhibition. *Sci Rep* **10**, 4566 (2020).
