# Supplementary material for: Locomotor deficits in a mouse model of ALS are paralleled by loss of V1-interneuron connections onto fast motor neurons
Source: Nat Commun. 2021 May 31;12:3251. doi: 10.1038/s41467-021-23224-7 (PMC8166981; doi:10.1038/s41467-021-23224-7)
Supplement: Supplementary file 3 — Description of Additional Supplementary Files [file 41467_2021_23224_MOESM3_ESM.pdf]

Description of Additional Supplementary Files

Supplementary Movie 1. Locomotor analysis of a SOD1G93A mouse at pre-symptomatic stage on postnatal day 49. Mouse was placed on a treadmill at a speed of 20 cm/s and filmed at 150 frames per second. Coloured circles in the video indicate digital markers used to extrapolate tracking information of areas of interest of the mouse (snout, torso, paws, and tail). Visualization of real time analysis shows profiles for speed, acceleration and cadence. Circular plot depicts phases of leftright alternation (perfect alternation being 180 degreed) and mean vector changes depending on the phase of the consecutive steps. Video is shown at 40 frames/s. Speed profile: treadmill belt speed is shown as a dashed blue line; final average speed of the mouse as a dotted orange line; average instantaneous speed from snout, torso and tail markers is quantified in real time at the top left corner and depicted as a solid blue line with standard deviation in grey. Acceleration profile: zero acceleration is shown as a dashed black line; instantaneous acceleration as a solid grey line; peak acceleration is marked with a red triangle; drag (in blue) and recovery (in orange) events are detected when there is a period of at least 0.25 s of continuous deceleration or acceleration, respectively. Cadence profile: relative position between left and right hind paws is depicted as a solid blue line; mean crossing point is shown as a dotted grey line and determines the estimated full stance-swing cycles, marked with red dots. Left-right alternation profile: phase differences between left and right hind limbs for each step are shown as dots, red for last step and blue for past ones; average phase difference is depicted as mean vector where directionality indicates average phase and radius measures the distribution of the angles (0 if uniformly distributed, 1 if all are equal).

Supplementary Movie 2. Analysis of the same SOD1G93A mouse shown in Supplementary video 1 after Onset of locomotor phenotype at postnatal day 63. Mouse was placed on a treadmill and filmed at 150 frames/s. Due to locomotor deficits the speed of the treadmill was reduced to 10 cm/s. Real time analysis of digital markers shows differences in speed, acceleration and cadence profiles; circular plot shows phases of left-right alternation. Video is shown at 40 frames/s. Speed, acceleration, cadence and left-right alternation profiles are shown as described in Supplementary Movie 1.

Supplementary Movie 3. Locomotor analysis of an age-matching En1cre;HoxB8FlipO;RC::Di mouse before Clozapine-N-Oxide administration. Panels represent speed, acceleration and cadence profiles of the mouse placed on a treadmill at a speed of 20 cm/s. Video was recorded at 150 frames/s. Phases of left-right alternation are depicted on the circular plot, and mean vector is represented by the arrow. Video is shown at 40 frames/s. Speed, acceleration, cadence and leftright alternation profiles are shown as described in Supplementary Movie 1.

Supplementary Movie 4. Analysis of the same En1cre;HoxB8FlipO;RC::Di mouse shown in Supplementary Movie 3 after administration of Clozapine-N-Oxide (CNO) (1 mg/kg) intraperitoneal. Fifteen minutes after silencing of spinal En1 interneurons, the mouse can walk on a treadmill only at a speed of 15 cm/s. Panels show changes in speed, acceleration and cadence. Left-right alternation depicted in circular plots remains unchanged after CNO administration. Video was recorded at 150 frames/s and is shown at 40 frames/s. Speed, acceleration, cadence and left-right alternation profiles are shown as described in Supplementary Movie 1.

Supplementary Movie 5. Locomotor analysis of a SOD1G93A;En1cre;HoxB8FlipO;RC::Di mouse at presymptomatic stage on postnatal day 49. Mouse was placed on a treadmill at 20 cm/s and recorded at 150 frames/s. Real time analysis of digital markers shows profiles for

speed, acceleration and cadence. Left-right alternation phases and mean vector are shown on the circular plot. Video is shown at 40 frames/s. Speed, acceleration, cadence and left-right alternation profiles are shown as described in Supplementary Movie 1.

Supplementary Movie 6. Locomotor analysis of the same SOD1G93A;En1cre;HoxB8FlipO;RC::Di mouse shown in Supplementary Movie 5 after Onset of locomotor phenotype at postnatal day 70, and before administration of Clozapine-N-Oxide. Mouse was placed on a treadmill and recorded at 150 frames/s. The speed of the treadmill was reduced to 15 cm/s due to locomotor deficits. Differences in speed, acceleration and cadence profiles from analysis of digital markers are shown in real time. Left-right alternation phases shown on the circular plot are maintained. Video is shown at 40 frames/s. Speed, acceleration, cadence and left-right alternation profiles are shown as described in Supplementary Movie 1.

Supplementary Movie 7. Locomotor analysis of the same SOD1G93A;En1cre;HoxB8FlipO;RC::Di mouse shown in Supplementary videos 5 and 6 at Onset of locomotor phenotype (postnatal day 70) and after administration of Clozapine-N-Oxide (CNO) (1 mg/kg) intraperitoneal. Fifteen minutes after silencing of spinal En1 interneurons, the mouse was placed on the treadmill and recorded at 150 frames/s. CNO administration had no further effect on locomotor deficits and the mouse could still perform at 15 cm/s as in Supplementary Movie 6. Real time speed, acceleration and cadence profiles are included. Left-right alternation also remains unchanged as shown on the circular plot. Video is shown at 40 frames/s. Speed, acceleration, cadence and left-right alternation profiles are shown as described in Supplementary Movie 1.
