# Supplementary material for: Locomotor deficits in a mouse model of ALS are paralleled by loss of V1-interneuron connections onto fast motor neurons
Source: Nat Commun. 2021 May 31;12:3251. doi: 10.1038/s41467-021-23224-7 (PMC8166981; doi:10.1038/s41467-021-23224-7)
Supplement: Supplementary file 11 — Reporting Summary [file 41467_2021_23224_MOESM11_ESM.pdf]

nature research

Corresponding author(s): Ole Kiehn
Ilary Allodi

Last updated by author(s): Mar 24, 2021

Reporting Summary

Nature Research wishes to improve the reproducibility of the work that we publish. This form provides structure for consistency and transparency in reporting. For further information on Nature Research policies, see our Editorial Policies and the Editorial Policy Checklist.

Statistics

For all statistical analyses, confirm that the following items are present in the figure legend, table legend, main text, or Methods section.

n/a	Confirmed	
☐	☒	The exact sample size (*n*) for each experimental group/condition, given as a discrete number and unit of measurement
☐	☒	A statement on whether measurements were taken from distinct samples or whether the same sample was measured repeatedly
☐	☒	The statistical test(s) used AND whether they are one- or two-sided<br>*Only common tests should be described solely by name; describe more complex techniques in the Methods section.*
☒	☐	A description of all covariates tested
☐	☒	A description of any assumptions or corrections, such as tests of normality and adjustment for multiple comparisons
☐	☒	A full description of the statistical parameters including central tendency (e.g. means) or other basic estimates (e.g. regression coefficient) AND variation (e.g. standard deviation) or associated estimates of uncertainty (e.g. confidence intervals)
☐	☒	For null hypothesis testing, the test statistic (e.g. *F*, *t*, *r*) with confidence intervals, effect sizes, degrees of freedom and *P* value noted<br>*Give P values as exact values whenever suitable.*
☒	☐	For Bayesian analysis, information on the choice of priors and Markov chain Monte Carlo settings
☒	☐	For hierarchical and complex designs, identification of the appropriate level for tests and full reporting of outcomes
☒	☐	Estimates of effect sizes (e.g. Cohen's *d*, Pearson's *r*), indicating how they were calculated

Our web collection on statistics for biologists contains articles on many of the points above.

Software and code

Policy information about availability of computer code

Data collection	DigiGait Imager software version 16-A6 from Mouse Specifics was used to capture videos. Tracking analysis of foot steps and limb joints was performed with DeepLabCut version 2.1.8.2. Anatomical pictures were acquired on Zeiss LSM 700 or 900 confocal microscopes using Zen Black version 14.0 and Zen Blue version 3.1 softwares, respectively.
Data analysis	Fiji software version 2.1.0/1.53c was used to quantify synaptic densities, while Zen Blue software version 3.2 was used for neuron and transcript quantifications. GraphPad Prism version 8.2.0 was used for regular statistical analysis. Python version 3.7 script was used to calculate locomotor parameters and circular statistics.<br>Code for locomotor analysis and figure generation is available on Github at https://github.com/kiehnlab/Locomotor-Allodi2021 or under DOI https://doi.org/10.5281/zenodo.4632328

For manuscripts utilizing custom algorithms or software that are central to the research but not yet described in published literature, software must be made available to editors and reviewers. We strongly encourage code deposition in a community repository (e.g. GitHub). See the Nature Research guidelines for submitting code & software for further information.

Data

Policy information about availability of data

All manuscripts must include a data availability statement. This statement should provide the following information, where applicable:

- Accession codes, unique identifiers, or web links for publicly available datasets
- A list of figures that have associated raw data
- A description of any restrictions on data availability

The raw data supporting the findings of the study are available from the corresponding authors upon reasonable request. Quantifications for statistical analysis are provided in the Source Data file.

Field-specific reporting

Please select the one below that is the best fit for your research. If you are not sure, read the appropriate sections before making your selection.

☒ Life sciences ☐ Behavioural & social sciences ☐ Ecological, evolutionary & environmental sciences

For a reference copy of the document with all sections, see nature.com/documents/nr-reporting-summary-flat.pdf

Life sciences study design

All studies must disclose on these points even when the disclosure is negative.

Sample size	No statistical methods were used to pre-determine sample sizes but our sample sizes are similar to those reported in previous publications (Cregg et al Nature Neuroscience 2020; Caggiano et al Nature 2018)
Data exclusions	No animal was excluded from the study.
Replication	All the experiments were performed at least in triplicates. For anatomical analysis:<br>- Synaptic density: 8-10 pictures per mouse per condition<br>- NMJ analysis: 16 pictures per mouse per condition<br>- RNAscope in situ hybridization: 8-10 tiled images per mouse per condition<br>For behavioral analysis three videos per mouse per condition per time point were recorded and analyzed.<br>In all cases, attempts at replication were successful.
Randomization	In all experiments, both anatomical and behavioral, mice were randomly allocated to different groups using a randomized block design.
Blinding	Data collection was performed blind to the conditions of the experiments, although in behavioral assessment ALS mice could be easily recognized at later stages. The analysis was automated and blind so that the experimenter had no influence on the outcome.

Reporting for specific materials, systems and methods

We require information from authors about some types of materials, experimental systems and methods used in many studies. Here, indicate whether each material, system or method listed is relevant to your study. If you are not sure if a list item applies to your research, read the appropriate section before selecting a response.

Materials & experimental systems

n/a	Involved in the study	
☐	☒	Antibodies
☒	☐	Eukaryotic cell lines
☒	☐	Palaeontology and archaeology
☐	☒	Animals and other organisms
☒	☐	Human research participants
☒	☐	Clinical data
☒	☐	Dual use research of concern

Methods

n/a	Involved in the study	
☒	☐	ChIP-seq
☒	☐	Flow cytometry
☒	☐	MRI-based neuroimaging

Antibodies

Antibodies used	For each antibody, we indicate: target - supplier, catalog number, lot number, host species (if applicable)<br>alfa-Bungarotoxin Alexa Fluor 488 - Invitrogen, B13422, 1903518<br>Neurofilament (NF-M) - DSHB, 2H3, 1/12/17, mouse<br>Synaptic vesicle glycoprotein 2A (SV2A) - DSHB, SV2, 10/12/17, mouse<br>Green Fluorescent Protein (GFP) - Abcam, Ab-13970, GR3190550-12, chicken<br>Red fluorescent protein (DsRed) - Clontech, 632496, 1509043, rabbit<br>Synaptophysin (SYN) Alexa Fluor 594 - Santa Cruz, sc-17750, B0118, mouse<br>Vesicular Glutamate Transporter 2 (VGLUT2) - Synaptic Systems, #135404, 2-38, guinea-pig<br>Matrix metallopeptidase 9 (MMP-9) - Sigma, M9570-100UG, MKBZ8513V, goat<br>Estrogen-related receptor beta (ErrBeta) - R&D, PP-H6705-00, A-2, mouse<br>Hemagglutinin (HA) tag - Sigma Aldrich, H6908, 057M4839V, rabbit<br>Neuronal nuclear protein (NeuN) - Millipore, ABN91, 3018826, chicken<br>Hoechst 33342 (blue fluorescent nucleic acid stain) - Invitrogen, H3570, 1915854<br>NeuroTrace 435/455 (blue fluorescent Nissl) - Invitrogen, N21479, 1846588<br>NeuroTrace 640/660 (deep-red fluorescent Nissl) - Invitrogen, N21483, 2172053<br>Alexa Fluor 488, 555, 568 & 647 - Invitrogen, A3272.../A3273.../A32931/A32849/A1107.../A31570, several combinations of excitation/emission wavelength and host/reactive species were used.

Validation

For each antibody, we indicate Antibody Registry ID, validation method, confirmed applications and reactivity by manufacturer, and/or references to example studies as available, as well as use in the present study.

Alfa-Bungarotoxin-AF488 - Applications: IF. Reactivity: nicotinic acetylcholine receptor of NMJs. (Allodi et al. 2016; Comley et al. 2016). Use: IHC, NMJ quantification.

NF-M - AB_2314897. Screened by indirect immunofluorescence by Dodd et al 1988. Applications: WB, IHC, IF. Reactivity: human, mouse, rat. (Allodi et al. 2016; Comley et al. 2016). Use: IHC, NMJ quantification.

SV2A - AB_2315387. Screened by solid-phase radioimmunoassay by Buckley & Kelly 1985. Applications: SB, IHC, IF. Reactivity: avian, bovine, fish, human, mouse, planaria, primate, rat, xenopus, zebrafish. (Allodi et al. 2016; Comley et al. 2016). Use: IHC, NMJ quantification.

GFP - AB_300798. Covered by Abpromise guarantee. Applications: WB, ICC, IF. Reactivity: cross-react with many fluorescent proteins derived from jellyfish Aequorea victoria. (Allodi et al. 2019). Use: IHC, synaptic density quantification.

DsRed - Quality and performance tested by WB by manufacturer. Applications: WB, IP, IL. Reactivity: various red fluorescent proteins in mammalian. (Caggiano et al. 2018). Use: IHC, synaptic density quantification.

SYN-AF594 - AB_628311. Applications: WB, ELISA, IP, IHC, IF. Reactivity: human, mouse, rat.
(Luo et al. 2019). Use: IHC, synaptic density quantification.

VGLUT2 - AB_887884. Validated in knock-out by Purrier et al. 2014. Applications: WB, IP, ICC, IHC, EM. Reactivity: mouse, rat.
(Ni et al. 2014). Use: IHC, synaptic density quantification.

MMP-9 - AB_1079397. Validated by immunoblotting and ELISA by manufacturer. Applications: WB, IP, IHC. Reactivity: mouse (10% cross-reactivity with recombinant human MMP-9). (Kaplan et al. 2014). Use: IHC, FF MN identification.

ErrBeta - AB_2100412. Applications: WB, ELISA, IP, IHC. Reactivity: human (also citations for mouse). (Enjin et al. 2010). Use: IHC, S MN identification.

HA-tag - AB_260070. Applications: WB, IP, IF. Reactivity: HA-tagged fusion proteins. (Li et al. 2019). Use: IHC, intersectional expression.

NeuN - AB_11205760. Applications: WB, IHC, ICC. Reactivity: mouse, rat (human also predicted based on sequence). (Magno et al. 2020). Use: IHC, intersectional expression.

Hoechst 33342 - Applications: IF. Reactivity: dsDNA. Use: IHC, counterstaining.

NT 435/455 & 640/660 - Applications: IF. Reactivity: Nissl Bodies. Use: IHC, counterstaining.

AF 488/555/568/647 - Applications: WB, IHC, ICC, IF. Reactivity specific to each AF. Use: IHC, secondary antibodies.

Animals and other organisms

Policy information about studies involving animals; ARRIVE guidelines recommended for reporting animal research

Laboratory animals

SOD1G93A (B6.Cg-Tg(SOD1-G93A)1Gur/J) (Jackson Laboratory stock no: #004435),wild-type C57BL6/J (Jackson Stock #000664), GlyT2GFP (Zeilhofer laboratory), HoxB8FlipO (Kiehn laboratory), En1Cre (Bikoff laboratory), R26R-EYFP (stock number: #006148 Jackson Laboratory) and RC::FPDi (Dymecki laboratory) animals and crosses hereoff were used. Experiments were performed with similar numbers of male and female mice.

For anatomical experiments, SOD1G93A mice and wild-type littermates were sacrificed at postnatal days P45, P63 and P84. In the case of SOD1G93A;En1Cre and En1Cre mice, intraspinal injections for viral delivery were performed at P42 and animals were sacrificed at P63 for analysis. Last, in the case of En1Cre;HoxB8FlipO;RC::FPDi mice, these were sacrificed one week after chemogenetic experiments.
For locomotor analysis, SOD1G93A mice and wild-type littermates were pre-trained at P42 and then tested weekly from P49 to P112. SOD1G93A;En1Cre;HoxB8FlipO;RC::FPDi mice were tested at these same timepoints, and chemogenetic experiments were performed at the time of onset of locomotor phenotype. Chemogenetic experiments on En1Cre;HoxB8FlipO;RC::FPDi mice were performed between P63 and P90.
For grip strength analysis, SOD1G93A mice and wild-type littermates were tested at P45, P60, P90, P120 and P140. In the case of chemogenetic experiments, same timepoints as for locomotor analysis.

Wild animals

No wild animals were used.

Field-collected samples

No field collected samples were used.

Ethics oversight

All animal experiments and procedures were approved by Dyreforsoegstilsynet (Danish Animal Inspectorate; ethical permit 2018-15-0201-01426) in Denmark and the local ethics committee at University of Copenhagen.

Note that full information on the approval of the study protocol must also be provided in the manuscript.
